# A urine extracellular vesicle circRNA classifier for detection of high-grade prostate cancer in patients with prostate-specific antigen 2–10 ng/mL at initial biopsy

**DOI:** 10.1186/s12943-021-01388-6

**Published:** 2021-07-23

**Authors:** Ya-Di He, Wen Tao, Tao He, Bang-Yu Wang, Xiu-Mei Tang, Liang-Ming Zhang, Zhen-Quan Wu, Wei-Ming Deng, Ling-Xiao Zhang, Chun-Kui Shao, Jing Zhou, Li-Min Rong, Xin Gao, Liao-Yuan Li

**Affiliations:** 1 Centre of Physical Examination, The Third Affiliated Hospital, Sun Yat-Sen University, Guangzhou, 510630 China; 2 Department of Urology, The Third Affiliated Hospital, Sun Yat-Sen University, Tianhe Road 600, Guangzhou, 510630 China; 3 Breast Surgery, The Third Affiliated Hospital, Sun Yat-Sen University, Guangzhou, 510630 China; 4 Department of Spine Surgery, The Third Affiliated Hospital, Sun Yat-Sen University, Guangzhou, 510630 China; 5 Department of Urology, Foshan First Municipal People’s Hospital, Sun Yat-Sen University, Foshan, 528000 China; 6 Department of Urology, The First Affiliated Hospital, University of South China, Hengyang, 421000 China; 7 Department of Urology, The First Affiliated Hospital, Hainan Medical College, Haikou, 570102 China; 8 Department of Pathology, The Third Affiliated Hospital, Sun Yat-Sen University, Guangzhou, 510630 China

**Keywords:** Prostate cancer, Circular RNA (circRNA), Diagnosis, Urine, Extracellular vesicle

## Abstract

The aim of this study was to identify a urine extracellular vesicle circular RNA (circRNA) classifier that could detect high-grade prostate cancer (PCa) of Grade Group (GG) 2 or greater. For this purpose, we used RNA sequencing to identify candidate circRNAs from urinary extracellular vesicles from 11 patients with high-grade PCa and 11 case-matched patients with benign prostatic hyperplasia. Using ddPCR in a training cohort (n = 263), we built a urine extracellular vesicle circRNA classifier (Ccirc, containing *circPDLIM5, circSCAF8*, *circPLXDC2*, *circSCAMP1*, and *circCCNT2*), which was evaluated in two independent cohorts (n = 497, n = 505). Ccirc showed higher accuracy than two standard of care risk calculators (RCs) (PCPT-RC 2.0 and ERSPC-RC) in both the training cohort and the validation cohorts. In all three cohorts, this novel urine extracellular vesicle circRNA classifier plus RCs was statistically more predictive than RCs alone for predicting ≥ GG2 PCa. This assay, which does not require precollection digital rectal examination nor special handling, is repeatable, noninvasive, and can be easily implemented as part of the basic clinical workflow.

**Supplementary Information:**

The online version contains supplementary material available at 10.1186/s12943-021-01388-6.

Prostate cancer (PCa) is the second most commonly diagnosed cancer in men [1].Currently, serum prostate-specific antigen (PSA) is the only widely used biomarker for PCa. Unfortunately, the low specificity (25–40%) of PSA in the so-called grey zone of PSA levels 2.0–10.0 ng/mL has resulted in a substantial increase in benign unnecessary biopsies along with the detection of clinically indolent disease [2]. Thus, there is an urgent need of more precise measures for identifying clinically significant PCa (high-grade PCa of Grade Group [GG] 2 or greater).

Many non-coding RNAs (eg, microRNAs, long non-coding RNAs, circular RNAs [circRNAs]) have been reported to play key roles in cancer progression, showing great potential to impact cancer diagnostics [3]. Specifically in PCa, 76,311 circRNAs have been identified through RNA sequencing of tumour specimens [4]. Interestingly, cancer-specific non-coding RNAs have been identified in extracellular vesicles [5]. Compared with linear RNAs, circRNAs have covalently linked ends of a single RNA molecular and appear a higher stability, which makes them to be more advantageous as potential molecular diagnostic markers [4]. In this study, we aimed to analyze circRNA expression profiles from urine-derived extracellular vesicles in high-grade PCa to develop a multi-circRNA-based classifier to detect high-grade PCa at initial biopsy. We evaluated the performance of this urine extracellular vesicle circRNA classifier in the training cohort, and validated externally it in two large independent cohorts. We also compared this assay performance with two standard of care risk calculators (RCs), Prostate Cancer Prevention Trial (PCPT)-RC 2.0 and European Randomized Study of Screening for Prostate Cancer (ERSPC)-RC [6, 7].

## Results and discussion

### Participants and clinicopathological characteristics

We collected 1265 first-catch non-digital rectal examination (DRE) urine samples (80–100 ml) from the three cohorts of 1265 eligible participants who had not been diagnosed with PCa, were aged 45 years or older, had a PSA 2.0–10.0 ng/mL, and scheduled for an initial prostate needle biopsy. Participants among these three cohorts (the training cohort, n = 263; validation cohort 1, n = 497; validation cohort 2, n = 505) were comparable with respect to general patient characteristics. (Fig S1, Table S2). All patients underwent at least 10-core transrectal ultrasound-guided biopsies and a central pathological review of all diagnostic biopsies. The GG was recorded according to the modified Gleason grading system using the International Society of Urological Pathology consensus [8]: GS2-6 = GG1, GS 3 + 4 = GG2, GS4 + 3 = GG3, GS8 = GG4 and GS9-10 = GG5. The total positive biopsy rate was 49.05% (21.67% GG1 and 27.38% ≥ GG2) for the training cohort, 40.64% (16.70% GG1 and 23.94% ≥ GG2) for validation cohort 1, and 47.52% (18.42% GG1 and 29.11% ≥ GG2) for validation cohort 2 (Table S2). This study was approved by the ethics committee at each study centre, and all participants provided written informed consent.

### RNA sequencing of urinary extracellular vesicles

In the discovery stage, we collected urine from 11 patients with high-grade PCa and 11 case-matched patients with benign prostatic hyperplasia (Table S1). The RNA sequencing was conducted by Illumina Hiseq X Ten system (Illumina, San Diego, CA, USA) on paired-end mode with length 150 bases following the vendor’s recommended protocol (Supplementary materials and methods). We defined the statistical criteria for selecting differentially expressed circRNAs using |fold changes|≥ 2.0 with p values < 0.05. We have deposited the RNA sequencing data reported in this study into the National Center for Biotechnology Information’s Gene Expression Omnibus (https://www.ncbi.nlm.nih.gov/geo/query/acc.cgi?acc=GSE147761).

**Fig. 1:**
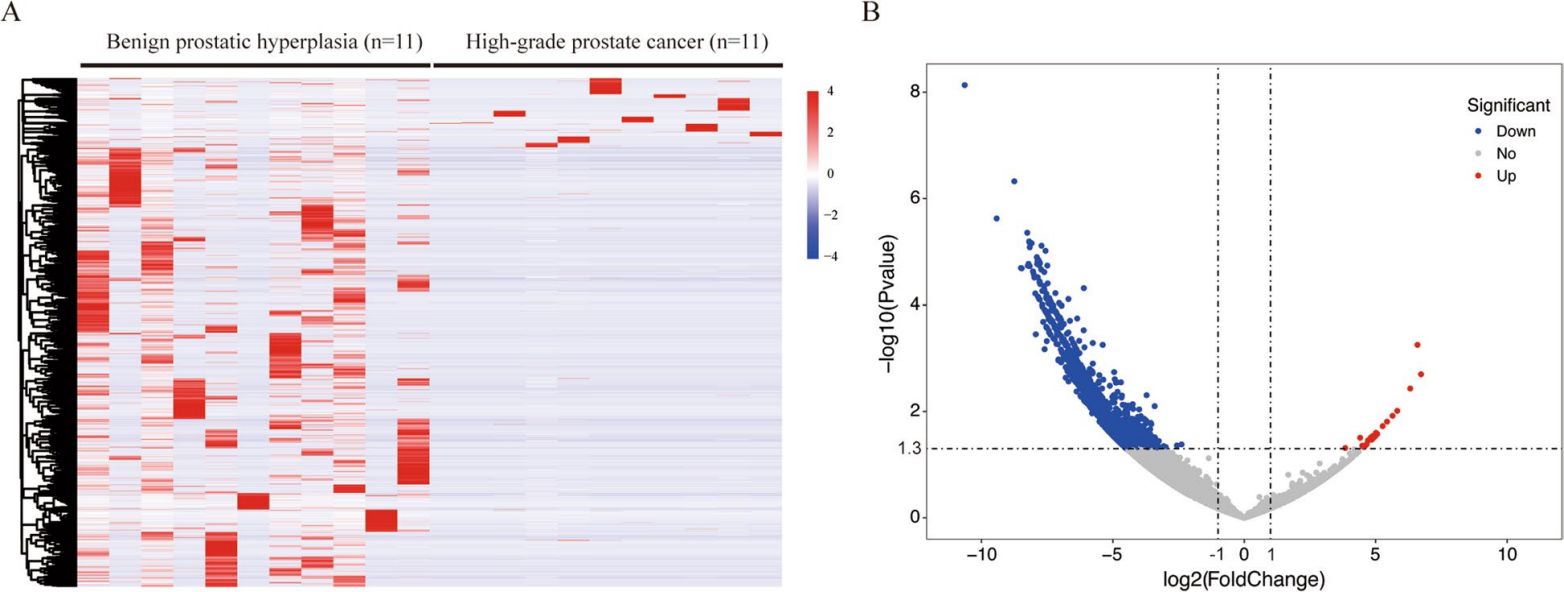
Differentially expressed urine extracellular vesicle circRNAs between individuals with benign prostatic hyperplasia and those with high-grade prostate cancer using RNA sequencing. (A) In the clustered heatmap, each column represents an individual sample, and each row represents an individual circRNA. The expression values were log2 transformed. (B) Eighteen candidate circRNAs displaying higher levels in patients with high-grade prostate cancer than those with benign prostatic hyperplasia according to the criteria using |fold changes|≥ 2.0 with p values < 0.05. circRNAs = circular RNAs

In total, we identified 2231 urine extracellular vesicle circRNAs that had significantly different levels between individuals with benign prostatic hyperplasia and those with high-grade PCa. Of these 2231 circRNAs, 18 circRNAs were upregulated in high-grade PCa and 2213 downregulated (Fig. 1, Table S3). These results are in accordance with previous reports that in general decreased circRNA levels were observed in PCa tumour samples compared to benign tissues [9]. Upregulated circRNAs may play oncogenic roles, promoting PCa cell proliferation, invasion, and migration, while downregulated circRNAs might have tumour suppressive functions, suppressing the survival, migration, invasion, and drug resistance of cancer cells [4, 9]. Because we were mainly interested in the potential markers which were practical and convenient to identify high-grade PCa in clinical practice, we focused on the 18 circRNAs that were increased in patients with high-grade PCa compared to patients with benign prostatic hyperplasia.

### Selection of candidate circRNAs and building a classifier

We confirmed the 18 increased circRNAs by digital droplet PCR (ddPCR) in 263 urine samples, which were collected from the training cohort of 263 participants (72 patients with high-grade PCa and 191 controls [including 134 patients with benign prostatic hyperplasia and 57 patients with GG1]) (Table S6-S8). Compared with those with benign prostatic hyperplasia, 18 of the candidate circRNAs were raised in the urine extracellular vesicles of patients with high-grade PCa. We used three models (linear discriminant analysis, support vector machine, and logistical regression) to build circRNA classifiers that could differentiate individuals with high-grade PCa from controls. Each circRNA combination with one model was considered as one classifier. Receiver operating characteristic (ROC) analysis was used to assess the area under receiver operating characteristic (AUC), accuracy, sensitivity, and specificity of the circRNA classifiers. Among all circRNA combinations with different models, a five-circRNA combination (which included *circPDLIM5, circSCAF8*, *circPLXDC2*, *circSCAMP1*, and *circCCNT2*) had high performance with all three models.

The ideal circRNA classifier, denoted as Ccirc, was constructed with logistical regression model, showing the largest AUC. (AUC, 0.820, Table S4, S5). The predicted probability of being diagnosed as high-grade PCa by Ccirc was calculated by:

$$\mathrm{Logit}\left[\mathrm{p}=\mathrm{high}-\mathrm{grade}\ \mathrm{PCa}\right]=-8.689+0.292\times \mathrm{circPDLIM}5+0.064\times \mathrm{circCCNT}2+0.053\times \mathrm{circSCAF}8+0.108\times \mathrm{circPLXDC}2+0.080\times \mathrm{circSCAMP}1.$$

Four circRNAs (*circSCAF8*, *circPLXDC2*, *circSCAMP1*, and *circCCNT2*) included in our Ccirc have been reported to be upregulated in PCa cells [4], suggesting that the altered expression of these circRNAs may contribute to PCa development.

### Validating the classifier

**Fig. 2:**
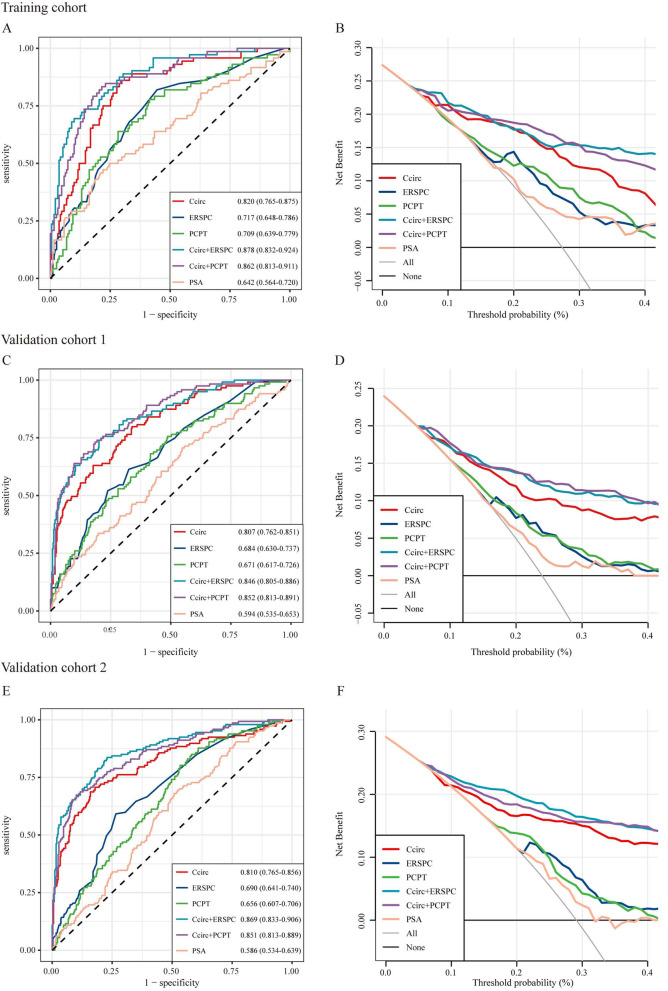
Performance of the Ccirc to detect high-grade PCa. Area under receiver operating characteristic curves (AUC) are shown to compare performances of the Ccirc in (A) the training cohort (n = 263), (C) the validation cohort 1 (n = 497), and (E) the validation cohort 2 (n = 505) with and without PCPT-RC, ERSPC-RC, and PSA alone. The corresponding net benefit analysis for the three cohorts is shown (B) for the training cohort, (D) for the validation cohort 1, and (F) for the validation cohort 2. Ccirc = classifier containing five circRNAs. PCa = prostate cancer. PSA = prostate-specific antigen. ERSPC-RC = European Randomized Study of Screening for Prostate Cancer risk calculator. PCPT-RC = Prostate Cancer Prevention Trial risk calculator

On comparing the performance of Ccirc with alternative models, Ccirc was superior to PCPT-RC 2.0, ERSPC-RC, and PSA alone for predicting ≥ GG2 PCa in both the training and validation cohorts (Fig. 2, Table S6). We then integrated this five-circRNA signature to two standard of care RCs, PCPT-RC 2.0 and ERSPC-RC, for predicting ≥ GG2 PCa. The addition of this five-circRNA signature achieved superior performance than did PCPT-RC 2.0 or ERSPC-RC alone, shown by a larger AUC (Fig. 2, Table S6). There were statistically significant differences in the median Ccirc values between patients with ≥ GG2 PCa and those with biopsy negative/GG1 PCa (p < 0.0001 for the training cohort, p < 0.0001 for the validation cohort 1, and p < 0.0001 for the validation cohort 2, Fig S4). Using a Ccirc cut-point of 7.539 copies/ml for predicting ≥ GG2 PCa in the training cohort, Ccirc showed an NPV of 93.01% with a sensitivity of 86.11% and would have avoided 50.57% of all biopsies (n = 263) or 69.63% of unnecessary, negative/GG1 biopsies (n = 191). This assay missed 10 of 72 (13.89%) ≥ GG2 cancers of which three were ≥ GG3. Similarly, the 7.539 copies/ml cut-point in the validation cohort 1 yielded a similar NPV of 87.50% and sensitivity of 66.39%, while avoiding 56.34% of biopsies, and 40 of 119 (33.61%) ≥ GG2 cancers were missed of which 17 were ≥ GG3. In the validation cohort 2, the same cut-point produced a NPV of 87.71% and sensitivity of 74.83%, while avoiding 52.28% of biopsies, and 37 of 147 (25.17%) ≥ GG2 cancers were missed of which 18 were ≥ GG3 (Table S7). The distributions of biopsy results from patients with different Ccirc values in the training and validation cohorts were also shown in waterfall plots (Fig S4).

This classifier (Ccirc) is designed to target the intended use population of patients with an equivocal PSA range (2.0–10.0 ng/mL) at initial biopsy, where standard of care variables (ie, age, race, family history, and PSA level) are less informative. Moreover, Ccirc alone had an AUC of 0.807–0.820, while four commercially available urinary biomarkers (Progensa PCA3, ExoDx prostate, SelectMDx, and MiPS) was 0.70–0.77, supporting robust performance of Ccirc. In addition, all these commercial urine tests, except for ExoDx prostate, need a DRE prior to collection [10, 11].

In the decision curve analyses (Fig. 2), compared with PSA and clinical-only models (ie, PCPT-RC 2.0, ERSPC-RC), Ccirc showed a higher net benefit across a wide range of decision threshold probabilities. In both the training and validation cohorts, Ccirc showed near-perfect calibration, with the predicted probabilities of high-grade PCa accurately, describing the true risk observed (Fig S3).

There is evolving understanding that GG2 and ≥ GG3 diseases have different cancer phenotypes. The ability to discriminate GG2 vs ≥ GG3 categories has important clinical implications for PCa management and prognosis [12]. We also investigated Ccirc value differences between GG2 category and ≥ GG3 category in the training and validation cohorts. We found that there were statistically significant differences in Ccirc values between these two categories, suggesting that this noninvasive assay may have the potential to differentiate individuals with GG2 PCa from those with ≥ GG3 disease (Fig S4). For patients with ≥ GG2 diseases, the minimum value of Ccirc was 5.41 copies/ml for the training cohort, 4.85 copies/ml for validation cohort 1, and 3.94 copies/ml for validation cohort 2. In addition, 161 urine samples were collected from 72 high-grade PCa patients in the training cohort after radical prostatectomy: 62 at 3 months, 54 at 6 months, and 45 at 12 months. Ccirc values significantly fell after radical surgery in all these patients. The median Ccirc value in urine before surgery was 8.51 (standard deviation: 1.19) copies/ml, and values dropped afterwards (7.99 [1.30] copies/ml at 3 months, p = 0.0211; 7.81 [1.10] copies/ml at 6 months, p = 0.0057; and 7.74 [1.17] copies/ml at 12 months, p = 0.0048, indicating that the increase of these circRNAs in urine extracellular vesicle might result from enhanced expression or secretion of circRNAs from PCa cells. (Fig S5) The disadvantage of this assay is possible technical inconveniences of handling large urine volumes (at least 80 ml) during RNA extraction.

## Conclusions

In summary, our data suggest that Ccirc could identify ≥ GG2 PCa in patients presenting for their initial biopsies with a PSA 2.0–10.0 ng/mL. It could improve two standard of care RCs (PCPT-RC 2.0 and ERSPC-RC) for predicting clinically significant PCa, with the potential to reduce unnecessary biopsies. In addition, this assay, which does not require precollection DRE nor special handling, is repeatable, noninvasive, and can be easily implemented as part of the basic clinical workflow.

## Supplementary Information

**Additional file 1.**

## Data Availability

All data in our study are available upon request.

## Abbreviations

AUC: Area under receiver operating characteristic
circRNA: Circular RNA
ddPCR: Digital droplet polymerase chain reaction
DRE: Digital rectal examination
ERSPC: European Randomized Study of Screening for Prostate Cancer
GG: Grade Group
NPV: Negative predictive value
PCa: Prostate cancer
PCPT: Prostate Cancer Prevention Trial
PSA: Prostate-specific antigen
RC: Risk calculator
ROC: Receiver operating characteristic
