# Supplementary material for: A urine extracellular vesicle circRNA classifier for detection of high-grade prostate cancer in patients with prostate-specific antigen 2–10 ng/mL at initial biopsy
Source: Mol Cancer. 2021 Jul 23;20:96. doi: 10.1186/s12943-021-01388-6 (PMC8299620; doi:10.1186/s12943-021-01388-6)
Supplement: Supplementary file 1 — Additional file 1. [file 12943_2021_1388_MOESM1_ESM.pdf]

A urine extracellular vesicle circRNA classifier for detection of high-grade prostate cancer in patients with prostate-specific antigen 2-10 ng/mL at initial biopsy

Supplementary Materials

Table of Contents

Supplementary methods……………………………………………………………………………2

Supplementary references……………………………………………………………………...8

Supplementary Tables

Table S1………………………………………………………………………………………………9

Table S2………………………………………………………………………………………………10

Table S3………………………………………………………………………………………………11

Table S4………………………………………………………………………………………………12

Table S5……………………………………………………………………………………………...13

Table S6………………………………………………………………………………………………14

Table S7………………………………………………………………………………………………15

Table S8………………………………………………………………………………………………16

Supplementary Figures

Figure S1……………………………………………………………………………………………..17

Figure S2……………………………………………………………………………………………..18

Figure S3……………………………………………………………………………………………..19

Figure S4……………………………………………………………………………………………..20

Figure S5………………………………………………………………………………………………21

Figure S6………………………………………………………………………………………………22

Figure S7………………………………………………………………………………………………23

Figure S8………………………………………………………………………………………………24

Supplementary methods

Participants

Participants were enrolled from The Third Affiliated Hospital of Sun Yat-sen University, Foshan First Municipal People's Hospital, The First Affiliated Hospital of University of South China, and The First Affiliated Hospital of Hainan Medical College. The urine used in the discovery stage was collected between June 1, 2016, and December 1, 2016, at The Third Affiliated Hospital of Sun Yat-sen University. The urine used in the training stage was collected between June 1, 2016, and June 30, 2020, at The Third Affiliated Hospital of Sun Yat-sen University. The validation stage had two independent cohorts. The first (cohort 1) enrolled participants from June 1, 2016, to March 31, 2020, at Foshan First Municipal People's Hospital. Cohort 2 was composed of participants at The First Affiliated Hospital of University of South China and The First Affiliated Hospital of Hainan Medical College enrolled from June 1, 2016, to June 30, 2020 (Fig S1, Table S2).

Central pathological review

In detail, H&E stained sections from individual sites representing all patients enrolled were sent to and centrally reviewed by a dedicated uropathologist (J. Z.) with 16 years of experience. The GG was recorded according to the modified Gleason grading system using the International Society of Urological Pathology consensus: GS2-6 = GG1, GS 3+ 4 = GG2, GS4 + 3 = GG3, GS8 = GG4 and GS9-10 = GG5.[1,2] We assessed the highest GG for each case. In cases in which the GG differed from that reported by the local pathologist, the samples were further reviewed by a third uropathologist (C.-K. S) who had 21 years of experience. The final review diagnosis was then decided by consensus. The uropathologists did not have any information regarding the urine extracellular vesicle circRNA assay results.

Statistical power analysis in discovery stage

To ensure that the sample size was competent enough to identify differences of clinical significance, the normalized data of candidate circRNAs with lowest fold change between 11 patients with high-grade prostate cancer (PCa) and 11 case-matched patients with benign prostatic hyperplasia (BPH) was used to statistical

power analysis.[3] The power which was calculated by 1-β was higher than 90%. The β was measured by means of formula as follows:

$$Z_\beta = \frac{\mu_{PCa} - \mu_{BPH}}{\sigma\sqrt{\frac{1}{n_{PCa}} + \frac{1}{n_{BPH}}}} - Z_{\alpha/2}$$

In this equation, $\alpha = 0.05$, $Z_{\alpha/2} = 1.96$, $n_{PCa} = 11$, $n_{BPH} = 11$, $\sigma = (\sigma_{PCa} + \sigma_{BPH})/2$.

The samples size was adequate to detect differences of clinical importance if the power was higher than 80%.

Urine sample preparation, extracellular vesicle isolation, and characterization

First-catch urine samples (80-100mL) were collected into two 50-mL Centrifuge tube (NEST Biotechnology Co.,Ltd) and stored without preservatives at 4°C for up to 3 days prior to filtering through a 0.8-μm syringe filter. Subsequently, samples were stored in 50-mL aliquots at -80°C until processed and analyzed centrally at The Core Lab Plat for Medical Science of Sun Yat-sen University.

Extracellular vesicles were isolated using ultracentrifugation and ExoQuick-TC extracellular vesicle precipitation solution (System Biosciences, Mountain View, CA) according to the manufacturer's instructions, with minor modifications. Briefly, the urinary samples were centrifuged at 3000 g for 15 min to remove cells and cell debris. Next, the supernatant was transferred to two 38.5-mL thinwall polyallomer centrifuge tube (Beckman Coulter, Fullerton, CA) and ultracentrifuged at 100,000 g for 2h at 4 °C (SW 70Ti rotor, Beckman Coulter, Brea, CA) to obtain extracellular vesicle-enriched pellet. Subsequently, the pellets were diluted to 5 mL in 1x phosphate-buffered saline and mixed with 1mL ExoQuick-TC by inverting or flicking the tube. After incubating overnight at 4°C with no rotation, the mixture was centrifuged at 1500 g for 30min at 4°C. The supernatant was discarded, and then centrifuged for another 5 min to remove residual fluid. Finally, the extracellular vesicles were resuspended with 1x phosphate-buffered saline stored at -80°C.

The isolated extracellular vesicles were characterized with FEI TECNAI G2 (FEI Ltd. Hillsboro, USA). Briefly, the isolated extracellular vesicles were fixed with 2% paraformaldehyde and adsorbed to copper grid. The copper grid was dried for 20 min at room temperature and then fixed with 1% Glutaraldehyde for 5 minutes. Next, the extracellular vesicles were stained with 4% uranyl acetate and dried for 5 minutes.

Subsequently, the extracellular vesicles were observed at 120 keV. For analysing the size and number of the isolated extracellular vesicles, Malvern Nanosight NS300 (Malvern Instruments Ltd., Malvern, UK) was used following the manufacturer's instructions. To analyze the protein markers of extracellular vesicles, Western blotting assays were performed using anti-CD9, anti-CD63, anti-HSP70, and anti-CD81 antibodies.[4,5]

RNA sequencing, the identification and annotation of circRNAs

In the discovery stage, we collected urine from 11 patients with high-grade PCa and 11 case-matched patients with benign prostatic hyperplasia (Table S1). Total RNA was extracted from urine-derived extracellular vesicles using miRNeasy Mini kit (Qiagen, Hilden, Germany) according to the manufacturer's instructions. The concentration and integrity of RNA was performed with Agilent 2100 bioanalyzer (Agilent, Santa Clara, CA, USA). For RNA sequencing, further purification of qualified total RNA was performed using RNAClean XP Kit (Beckman Coulter, Inc. Kraemer Boulevard Brea, CA, USA) and RNase-Free DNase Set (Qiagen, GmBH, Germany).

The total RNA was treated with Ribo-Zero Gold rRNA Removal Kit (Human/Mouse/Rat) (illumine, MRZG12324) to delete rRNA according to the manufacturer’s instructions. Next, strand-specific libraries were constructed using NEBNext Ultra II RNA Library Prep Kit (NEB, E7770) following the manufacturer’s instructions. In brief, the RNAs were fragmented using fragmentation buffer and then reverse transcribed into first-strand cDNA and second-strand cDNA in orderly. Then, the cDNA fragments were purified, subjected to end-repair, and modified to add poly (A) to the 3’ end. The sequencing adapters were ligated to the cDNA fragments. The double strand cDNA was digested by Second Strand Enzyme Mix (Nugen, S01531) to remove the second-strand cDNA before sequencing. The purified ligation products were performed for PCR amplification, then the amplification products of cDNA were purified with AMPure XP beads (Beckman, A63881). The quantitativeness of library and the distribution of fragment were assessed with Qubit HS DNA kit (life, Q32854) and 2100 DNA high sensitivity kit (Agilent, 5067-4626), respectively. The RNA-seq was conducted by Illumina Hiseq X Ten system (Illumina, San Diego, CA, USA) on paired-end (PE) mode with length 150 bases following the vendor’s recommended protocol.

For the identification and annotation of circRNAs, the raw sequencing reads were subjected to trimming process with SOAPnuke (v.1.5.2). High-quality reads were aligned to the Homo sapiens reference genome (GRCH37/hg19) that was obtained from UCSC genome browser (http://genome.ucsc.edu/) using bowtie2 (v.2.2.9)[6] and Tophat2 (v.2.1.0).[7] The resulted sequence alignment files (bam formatted file) were then sorted by reference position as required by SAM tools (v.2.6.2).

CircRNAs were identified and quantified by find_circ(v.1.2)[8] and CIRCexplorer2 (v.2.3.2).[9] The identified circRNAs were then annotated with circBase database that contains data from studies of large-scale circRNAs identification published to date.[10] We compared circRNAs expression profiles in urine extracellular vesicles across 22 case-matched patients with high-grade PCa or with benign prostatic hyperplasia using the DESeq2 (v.1.22.2) package of R (v.3.1.2).[11] We defined the statistical criteria for selecting differentially expressed circRNAs using $|\text{fold changes}| \geq 2.0$ with p values < 0.05. We have deposited the RNA sequencing data reported in this study into the National Center for Biotechnology Information's Gene Expression Omnibus (GSE147761).

Reverse transcription

The extracellular vesicle RNA was reversely transcribed to cDNA with random primers using PrimeScript RT reagent Kit, Perfect Real Time (TaKaRa, Dalian, China) following the manufacturer's instructions. To minimize inter-sample variability, we used consistent volumes throughout the entire process, starting from urine to the final digital droplet PCR (ddPCR) amplification.

Construction of pUC57-circRNA vector

The standard curves were prepared using pUC57-circRNA vector constructed by Sangon Biotech Company, Ltd (Shanghai, China). Briefly, the five PCR products that were chemically synthesized by sequences of PCR products and primers were cloned into pUC57 vector, respectively. The initial concentrations by dilution of (*circPDLIM5, circSCAF8*, *circPLXDC2*, *circSCAMP1*, and *circCCNT2*) were $8.24\text{x}10^9$ copies/μL, $7.29\text{x}10^9$ copies/μL, $8.32\text{x}10^9$ copies/μL, $6.82\text{x}10^9$ copies/μL, and $8.79\text{x}10^9$ copies/μL, respectively (Figure S7,8).

Digital droplet polymerase chain reaction (ddPCR) analysis

We used ddPCR EvaGreen Supermix (Bio-Rad, Foster City, CA, USA) on the QX200 Droplet System. The primers used are listed in Table S6. The reactions were assembled into each well according to the following protocol: 2 μl cDNA, 10 μl QX200 EvaGreen ddPCR Supermix (Bio-Rad), 4 μM of each primer and nuclease-free water up to 20 μl. A no template control, where nuclease-free water was added instead of cDNA samples, was set. The droplets generation procedure followed the manufacturer's instruction. Each 70 μl QX200 Droplet generation oil and 20 μl ddPCR reaction respectively were added into the 8-channel droplet generation cartridge and the cartridge was loaded in the QX200™ Droplet Generator. Subsequently, the 40 μl of resulting droplets solution was then transferred into a 96-well PCR plate. The cycling conditions were as follows: hot-start at 95°C for 5 min; followed by 45 cycles of 95°C for 30 s, 64°C for 1 min; then, a signal stabilization step at 4°C for 5 min and 90°C for 5 min, finally holding at 4°C. Droplets were detected on the QX200 Droplet Reader (Bio-Rad) and analysed by QuantaSoft software (Bio-Rad). The resulting copies per microliter of reaction were the numbers exported by the software.

The laboratory investigators have no information regarding patients' clinical and outcome data and researchers at all institutions were masked to circRNAs testing results. After all samples had been tested by the same central laboratory (The Core Lab Plat for Medical Science of Sun Yat-sen University), these blinded data were released to and compared with biopsy outcomes.

Classifier construction in the training stage

The levels of 18 candidate circRNAs identified in the discovery stage were analyzed in the training cohort. We used three different algorithms to select the most significant circRNAs for classifying ≥GG2 diseases and biopsy negative/GG1 diseases. The exhaustive algorithm was applied to create all possible combinations of 18 candidate circRNAs and the bootstrapping resampling (boot) was used to evaluate the performance of each circRNA combination with either of three classification models. First, we performed the linear discriminant analysis (LDA)[12] to identify a set of 7 circRNAs. Second, we used the support vector machine (SVM) algorithm[13] and selected a set of 16 circRNAs. Finally, the logistical regression (LR)[14] was applied to identify another set of 8 circRNAs. After combining the circRNAs selected by these three different algorithms, 5 circRNAs (*circPDLIM5, circSCAF8*, *circPLXDC2*,

circSCAMP1, and *circCCNT2*) were identified, being selected simultaneously by the three algorithms.

The receiver operating characteristic (ROC) analysis was used to assess the performance of this five-circRNA combination with all three algorithms, including area under the ROC curve (AUC), accuracy, sensitivity, and specificity. Each circRNA combination with one algorithm is defined as a classifier. The ideal classifier with the largest AUC and accuracy was then externally validated in two independent cohorts.

All statistical analyses were conducted with SPSS (version 20.0; SPSS Inc, Chicago, IL, USA) or R software version 3.4.3 (R Foundation for Statistical Computing, Vienna, Austria).

Supplementary references

1 Epstein JI. Prostate cancer grading: a decade after the 2005 modified system. *Mod Pathol* 2018; 31(S1): S47-63.

2 Epstein JI, Amin MB, Reuter VE, Humphrey PA. Contemporary Gleason Grading of Prostatic Carcinoma: An Update With Discussion on Practical Issues to Implement the 2014 International Society of Urological Pathology (ISUP) Consensus Conference on Gleason Grading of Prostatic Carcinoma. *Am J Surg Pathol* 2017; 41(4): e1-e7.

3 Lachin JM. Introduction to sample size determination and power analysis for clinical trials. *Control Clin Trials* 1981; **2**: 93-113.

4 Vicencio JM, Yellon DM, Sivaraman V, et al. Plasma exosomes protect the myocardium from ischemia-reperfusion injury. *J Am Coll Cardiol* 2015; **65**: 1525-36.

5 Kowal J, Arras G, Colombo M, et al. Proteomic comparison defines novel markers to characterize heterogeneous populations of extracellular vesicle subtypes. *Proc Natl Acad Sci U S A* 2016; **113**: E968-77.

6 Langmead B, Salzberg SL. Fast gapped-read alignment with Bowtie 2. *Nat Methods* 2012; 9(4): 357-9.

7 Kim D, Pertea G, Trapnell C, Pimentel H, Kelley R, Salzberg SL. TopHat2: accurate alignment of transcriptomes in the presence of insertions, deletions and gene fusions. *Genome Biol* 2013; 14(4): R36.

8 Memczak S, Jens M, Elefsinioti A, et al. Circular RNAs are a large class of animal RNAs with regulatory potency. *Nature* 2013; 495(7441): 333-8.

9 Zhang XO, Wang HB, Zhang Y, Lu X, Chen LL, Yang L. Complementary sequence-mediated exon circularization. *Cell* 2014; 159(1): 134-47.

10 Glazar P, Papavasileiou P, Rajewsky N. circBase: a database for circular RNAs. *RNA* 2014; 20(11): 1666-70.

11 Love MI, Huber W, Anders S. Moderated estimation of fold change and dispersion for RNA-seq data with DESeq2. *Genome Biol* 2014; 15(12): 550.

12 Fisher R. The use of multiple measures in taxonomic problems. *Ann. Eugenics* 1936; **7:** 179-88.

13 Noble WS. What is a support vector machine? *Nat Biotechnol* 2006; **24:** 1565-67.

14 Sperandei S. Understanding logistic regression analysis. *Biochem Med* 2014; **24:** 12-18.

***Table S1:* Clinicopathological characteristics of participants in the discovery cohort**

	Benign prostatic hyperplasia (n=11)	High-grade PCa (n=11)	p value
Age, years			
Median (IQR)	64.0 (62.5-67.0)	69.0 (62.5-73.0)	0.264†
PSA, ng/mL			
Median (IQR)	3.96 (3.44-5.22)	5.61 (4.24-6.37)	0.065†
Family history, n			-
Yes	0	0	
No	11	11	
Grade Group, n			-
Benign	11	-	
GG 1 (ISUP1, GS3 + 3)	-	-	
GG 2 (ISUP2, GS3 + 4)	-	2	
GG 3 (ISUP3, GS4 + 3)	-	3	
GG 4 (ISUP4, GS8)	-	4	
GG 5 (ISUP5, >GS8)	-	2	

PCa=prostate cancer. IQR=interquartile range. PSA=prostate-specific antigen. GG=Grade Group. ISUP=International Society of Urological Pathology. GS=Gleason score. †Mann-Whitney U test

***Table S2*: Clinicopathological characteristics of participants in the training and validation cohorts**

	Training cohort	Validation cohort 1	Validation cohort 2
Total	263	497	505
Age, years, median, IQR	69 (63-75)	70 (62-78)	69 (59-78)
PSA, ng/mL, median, IQR	5.59 (3.39-7.34)	5.33 (3.96-7.19)	5.21 (3.86-7.72)
Family history, n (%)			
Yes	78 (29.66)	94 (18.91)	83 (16.44)
No	185 (70.34)	384 (77.26)	393 (77.82)
NA	-	19 (3.82)	29 (5.74)
DRE, n (%)			
Nonsuspicious	184 (69.96)	314 (63.18)	368 (72.87)
Suspicious	79 (30.04)	103 (20.72)	87 (17.23)
NA	-	80 (16.10)	50 (9.90)
Volume, ml, median, IQR	41.3 (30.4-51.7)	40.4 (28-55.3)	41.4 (30.2-55.4)
Grade Group, n (%)			
Benign	134 (50.95)	295 (59.36)	265 (52.48)
GG 1 (ISUP1, GS3 + 3)	57 (21.67)	83 (16.70)	93 (18.42)
GG 2 (ISUP2, GS3 + 4)	39 (14.83)	59 (11.87)	82 (16.24)
GG 3 (ISUP3, GS4 + 3)	14 (5.32)	29 (5.84)	28 (5.54)
GG 4 (ISUP4, GS8)	11 (4.18)	19 (3.82)	21 (4.16)
GG 5 (ISUP5, >GS8)	8 (3.04)	12 (2.41)	16 (3.17)
≥GG 2 (ISUP2, GS3 + 4)	72 (27.38)	119 (23.94)	147 (29.11)

IQR=interquartile range. PSA=prostate-specific antigen. NA=not available. DRE=digital rectal examination. GG=Grade Group. ISUP=International Society of Urological Pathology. GS=Gleason score.

Table S3: **Eighteen candidate circRNAs displaying higher levels in patients with high-grade prostate cancer than those with benign prostatic hyperplasia in the discovery cohort**

circRNA ID	Gene symbol	Spliced length （bp）	Position	Strand	log(Fold change)	p value
hsa_circ_0127850	ADAMTS19	565	chr5: 128861976-128932357	(+)	6.73	0.0020
hsa_circ_0049335	DNM2	294	chr19: 10906047-10909248	(+)	6.32	0.0037
hsa_circ_0081867	ATXN7L1	847	chr7: 105278799-105305735	(-)	5.82	0.0097
hsa_circ_0004028	PDLIM5	1012	chr4: 95444874-95539342	(+)	5.64	0.0120
hsa_circ_0003169	TCONS_l2_00026703	273	chr7: 76713203-76728268	(-)	5.43	0.0155
hsa_circ_0008475	ARPC2	281	chr2:219103386-219104207	(+)	5.06	0.0265
hsa_circ_0078367	SCAF8	1390	chr6:155095122-155131342	(+)	4.97	0.0294
hsa_circ_0136575	C8orf40	263	chr8:42401611-42403923	(+)	4.95	0.0301
hsa_circ_0126027	PPARGC1A	1142	chr4:23814369-23816228	(-)	4.90	0.0320
hsa_circ_0126176	PCDH7	313	chr4:30921774-30951837	(+)	4.89	0.0287
hsa_circ_0131073	SYNE1	503	chr6:152557241-152560841	(-)	4.84	0.0340
hsa_circ_0017924	PLXDC2	342	chr10:20432223-20453496	(+)	4.81	0.0313
hsa_circ_0127852	ADAMTS19	494	chr5:129030412-129040084	(+)	4.71	0.0349
hsa_circ_0095658	NAP1L4	433	chr11: 2985909-2993473	(-)	4.63	0.0429
hsa_circ_0073111	SCAMP1	575	chr5: 77684660-77717784	(+)	4.55	0.0468
hsa_circ_0040507	WDR59	370	chr16:74926381-74937998	(-)	4.49	0.0438
hsa_circ_0056536	CCNT2	190	chr2:135694410-135696632	(+)	4.41	0.0313
hsa_circ_0134483	-	1697	chr7: 61969420-61971117	(-)	3.85	0.0483

circRNAs=circular RNAs.

Table S4: **The performance of the ideal circRNA combination in the training cohort**

**Model**		**Cutoff (copies/ml)**	**Performances**			
			**AUC (95% CI)**	**Accuracy (%)**	**Sensitivity (%)**	**Specificity (%)**
Individual circRNA	circPDLIM5	6.43	0.720(0.662-0.774)	58.17	88.89	46.60
	circSCAF8	21.86	0.660(0.600-0.717)	61.22	72.22	57.07
	circPLXDC2	17.05	0.611(0.549-0.671)	72.62	40.28	85.34
	circSCAMP1	11.48	0.593(0.531-0.653)	53.99	76.39	45.55
	circCCNT2	20.28	0.724(0.666-0.777)	61.22	81.94	53.93
Five-circRNA combination	SVM	-	0.813(0.760-0.858)	73.00	87.50	68.06
	LDA	-	0.724(0.666-0.777)	61.22	81.94	53.93
	LR	-	0.820(0.768-0.865)	73.76	86.11	70.16

circRNA=circular RNA. AUC=area under receiver operating characteristic. CI=confidence interval. SVM=support vector machine. LDA=linear discriminant analysis. LR=logistical regression.

Table S5: **Coefficients for the final logistical regression equation of Ccirc**

	**coef**	**exp(coef)**	**se(coef)**	**wald**	**p value**
circPDLIM5	0.292	1.339	0.069	17.947	0.00002
circSCAF8	0.053	1.055	0.026	4.193	0.04058
circPLXDC2	0.108	1.114	0.039	7.833	0.00513
circSCAMP1	0.080	1.084	0.032	6.175	0.01295
circCCNT2	0.064	1.066	0.016	16.266	0.00006
Intercept	-8.689	0.0002	1.261	47.509	<0.00001

Ccirc= classifier containing five circRNAs.

Table *S6*: Performance comparison of the Ccirc to alternative models

Test/model	Training cohort (n=263)		Validation cohort 1 (n=497)		Validation cohort 2 (n=505)	
	AUC (95% CI)	p value	AUC (95% CI)	p value	AUC (95% CI)	p value
Ccirc	0.820 (0.765-0.875)	-	0.807 (0.762-0.851)	-	0.810 (0.765-0.856)	
ERSPC-RC	0.717 (0.648-0.786)	0.0202	0.684 (0.630-0.737)	<0.0001	0.690 (0.641-0.740)	0.0006
PCPT-RC	0.709 (0.639-0.779)	0.0160	0.671 (0.617-0.726)	<0.0001	0.656 (0.607-0.706)	<0.0001
Ccirc+ ERSPC-RC	0.878 (0.832-0.924)	0.0066	0.846 (0.805-0.886)	0.0023	0.869 (0.833-0.906)	<0.0001
Ccirc+ PCPT-RC	0.862 (0.813-0.911)	0.0301	0.852 (0.813-0.891)	0.0002	0.851 (0.813-0.889)	0.0007
PSA	0.642 (0.564-0.720)	0.0003	0.594 (0.535-0.653)	<0.0001	0.586 (0.534-0.639)	<0.0001

Ccirc=classifier containing five circRNAs. AUC=area under receiver operating characteristic. CI=confidence interval. PSA=prostate-specific antigen. ERSPC-RC=European Randomized Study of Screening for Prostate Cancer risk calculator. PCPT-RC=Prostate Cancer Prevention Trial risk calculator.

Table S7: **Performance of the Ccirc in the training and validation cohorts**

	**Ccirc ≥ cut-point**	**Ccirc < cut-point**	**Total**	**Performance, %**	**(95%CI)**
**Training cohort**					
Biopsy Positive/ ≥ GG2	62	10	72	Sensitivity, 86.11%	(75.48-92.78)
Biopsy Negative/GG1	58	133	191	Specificity, 69.63%	(62.51-75.96)
Total	120	143	263	PPV, 51.67%	(42.41-60.82)
				NPV, 93.01%	(87.18-96.41)
**Validation cohort 1**					
Biopsy Positive/ ≥ GG2	79	40	119	Sensitivity, 66.39%	(57.07-74.63)
Biopsy Negative/GG1	98	280	378	Specificity, 74.07%	(69.29-78.36)
Total	177	320	497	PPV, 44.63%	(37.23-52.27)
				NPV, 87.50%	(83.25-90.82)
**Validation cohort 2**					
Biopsy Positive/ ≥ GG2	110	37	147	Sensitivity, 74.83%	(66.89-81.45)
Biopsy Negative/GG1	94	264	358	Specificity, 73.74%	(68.80-78.16)
Total	204	301	505	PPV, 53.92%	(46.83-60.86)
				NPV, 87.71%	(83.33-91.09)

Ccirc=classifier containing five circRNAs. CI=confidence interval. GG=Grade Group. NPV=negative predictive value. PPV = positive predictive value.

Table S8: **Sequences of primers used for digital droplet PCR in this study**

CircRNA ID	Gene symbol	Length (bp)	Forward(5'-3')	Reverse(5'-3')
hsa_circ_0049335	DNM2	107	CCGAGAAGCTCAGTTCCTAC	GTCAAACTCCATCTGGTCCT
hsa_circ_0004028	**PDLIM5**	106	GTTACCAGCCTCACAGCTGC	GCCGCCATCTTTTAGCTCCT
hsa_circ_0008475	ARPC2	99	GGTGTTCATGCAGGATACAA	TCGCTTCAACATGCCAGCTT
hsa_circ_0126027	PPARGC1A	118	GCAGAAGGCAATTGCCTAAC	ATGGTGGTGGCACCACAGTC
hsa_circ_0056536	**CCNT2**	128	GAGCCACTGCTGGATACTAA	CCACTTTTGCAGCCAAAAAT
hsa_circ_0127850	ADAMTS19	108	CTTGAGTGGAATGTGTAGTG	GTCTTCCTTTATTTGTGACC
hsa_circ_0081867	ATXN7L1	126	ACTCTGTACTTCCTAGAGAG	ATGGCCGCTGAGACAGGCTT
hsa_circ_0003169	TCONS_l2_00026703	110	GTTGTAACAGTGACTGGTAA	ACATCTATGGCACCCTGATT
hsa_circ_0078367	**SCAF8**	92	AGTGAAAGGAGAGCCAGAGA	TCAGGGAATACAACACTTAG
hsa_circ_0136575	C8orf40	108	GTGTGCCACCTACAGAGGAA	CCAGCCATGGGGAAATGGAA
hsa_circ_0126176	PCDH7	100	CTCCATTAATCAACAGCCAT	CCCGCTGTCATAGCAACTCT
hsa_circ_0131073	SYNE1	95	GAGAGTGCTGGCTAAGTAAC	GGCCCTGAAAGTATTCCTGG
hsa_circ_0017924	**PLXDC2**	152	CCAACAAATTCCCAGTTTCA	CCACAAGTGCTGTGCCATTA
hsa_circ_0127852	ADAMTS19	136	TCGGACCGTTAGATGTACCA	TTGTCTTCCTTTCTCCTTAG
hsa_circ_0095658	NAP1L4	143	CCCTGGACAGCCTATGTTTA	CTGGTATAGCGCTGCATACT
hsa_circ_0073111	**SCAMP1**	120	GATTCTGCAAGAGCGGTTGA	GTGTAACTGATGGATCCTGA
hsa_circ_0040507	WDR59	113	CGCTGGGAGAGCTGTACATA	GCTGAGAGAGATCTGGACAA
hsa_circ_0134483	-	105	CTCTCTAAAGCAAGGTTCAA	TTGCAAACCGGATTTCTTCA

circRNA=circular RNA.

Figure S1

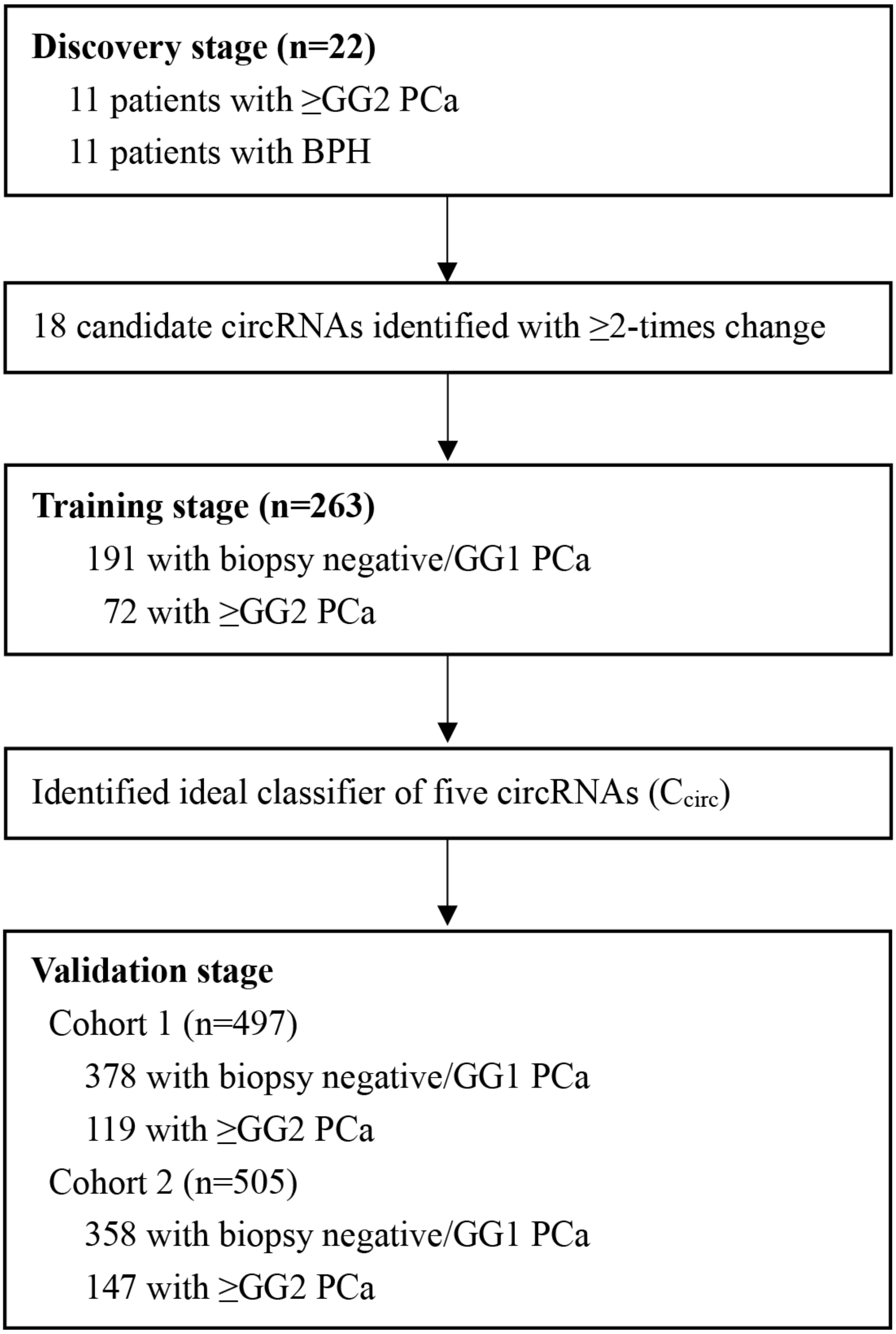

Figure S1: **Study flow**

GG=Grade Group. PCa=prostate cancer. BPH=benign prostatic hyperplasia. circRNAs=circular RNAs. Ccirc= classifier containing five circRNAs.

Figure S2

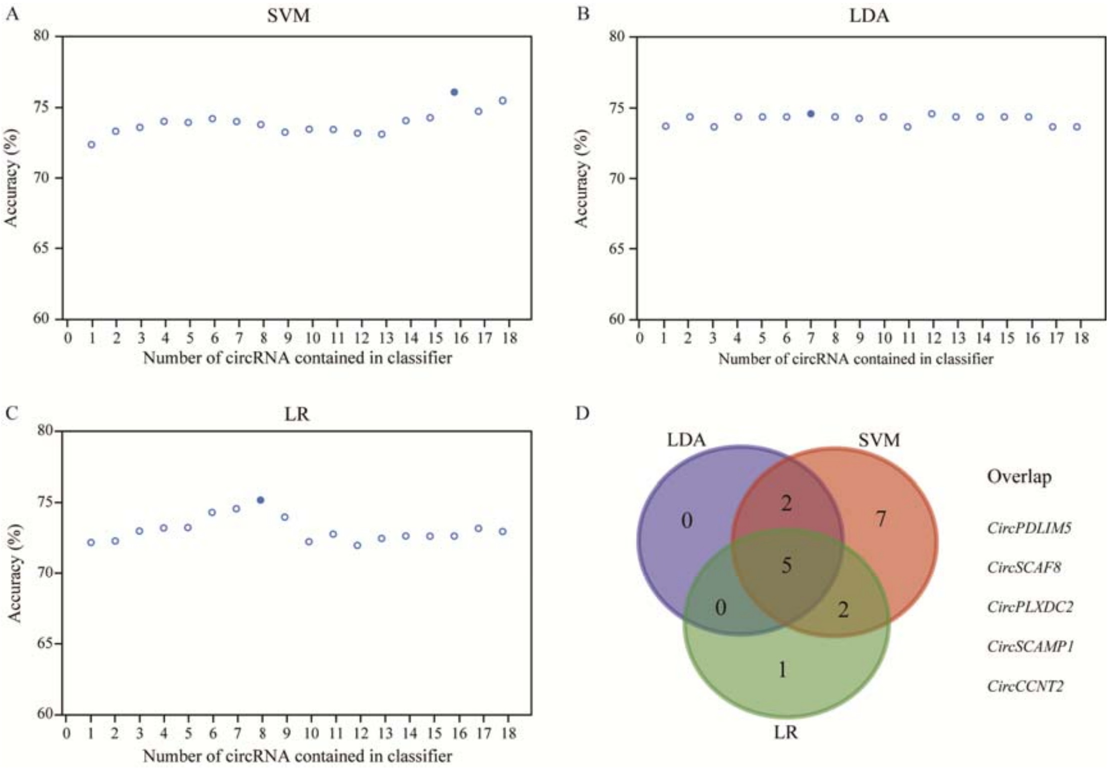

***Figure S2:* The performance of urine extracellular vesicle circRNA classifiers to detect high-grade PCa in three classification models**

(A-C) Three models, including SVM, LDA, and LR models, were applied to construct circRNA classifiers. The bootstrapping resampling (boot) was used to evaluate the performance of classifiers to detect high-grade PCa. The *x*-axis denotes the number of circRNAs contained in the classifier, while the *y*-axis indicates the performance of the classifiers. In each group with the same circRNA number, only the classifier showing the best accuracy is presented. (D) Five circRNAs (*circPDLIM5*, *circSCAF8*, *circPLXDC2*, *circSCAMP1*, and *circCCNT2*) were identified, being selected simultaneously by all three models. circRNA=circular RNA. PCa=prostate cancer. SVM=support vector machine. LDA=linear discriminant analysis. LR=logistical regression.

Figure S3

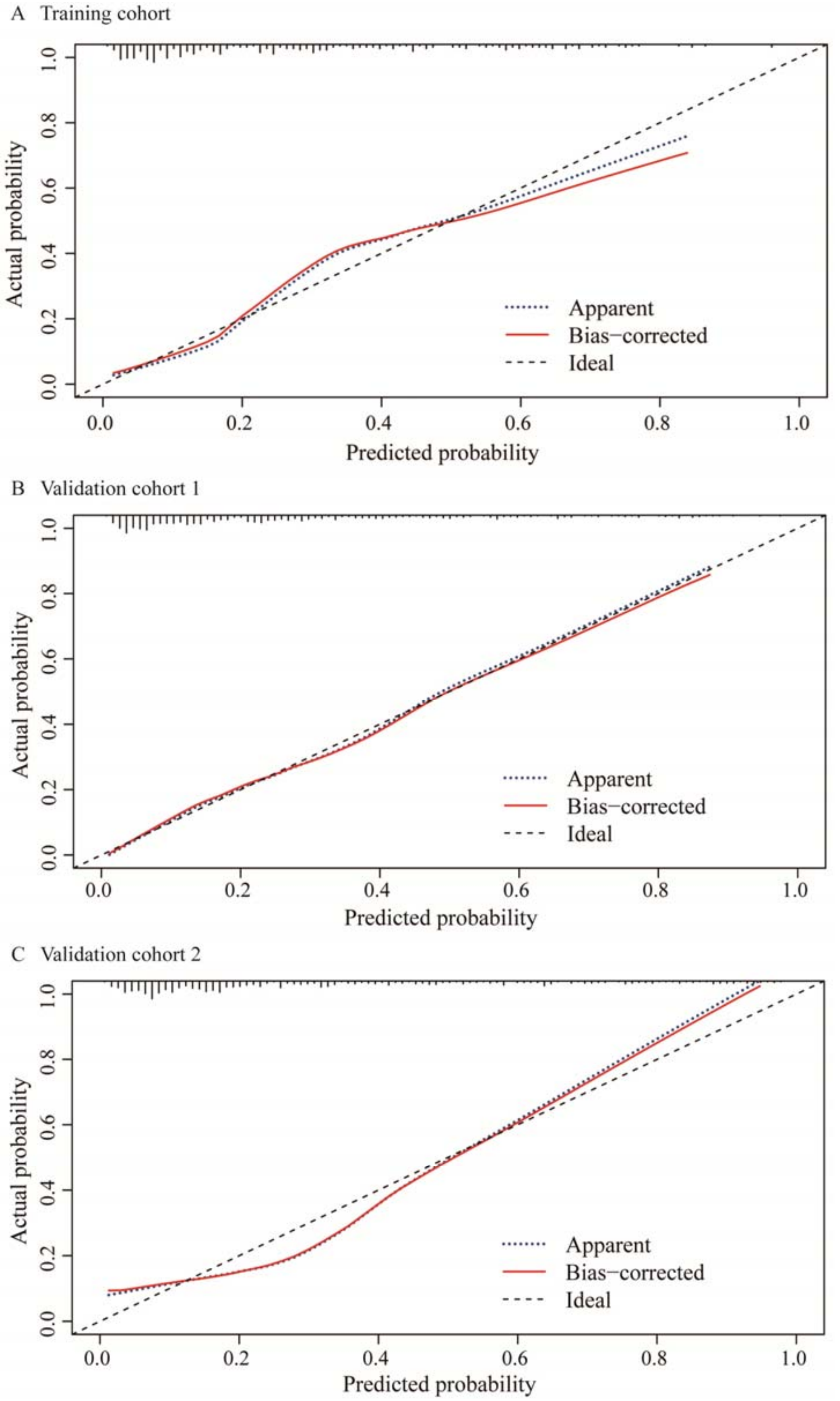

Figure S3: **Calibration plots of the Ccirc to detect high-grade PCa**

Calibration plots showing predicted vs actual high-grade PCa detected using the Ccirc in the training cohort (A), the validation cohort 1 (B), and the validation cohort 2 (C). Ccirc=classifier containing five circRNAs. PCa=prostate cancer.

Figure S4

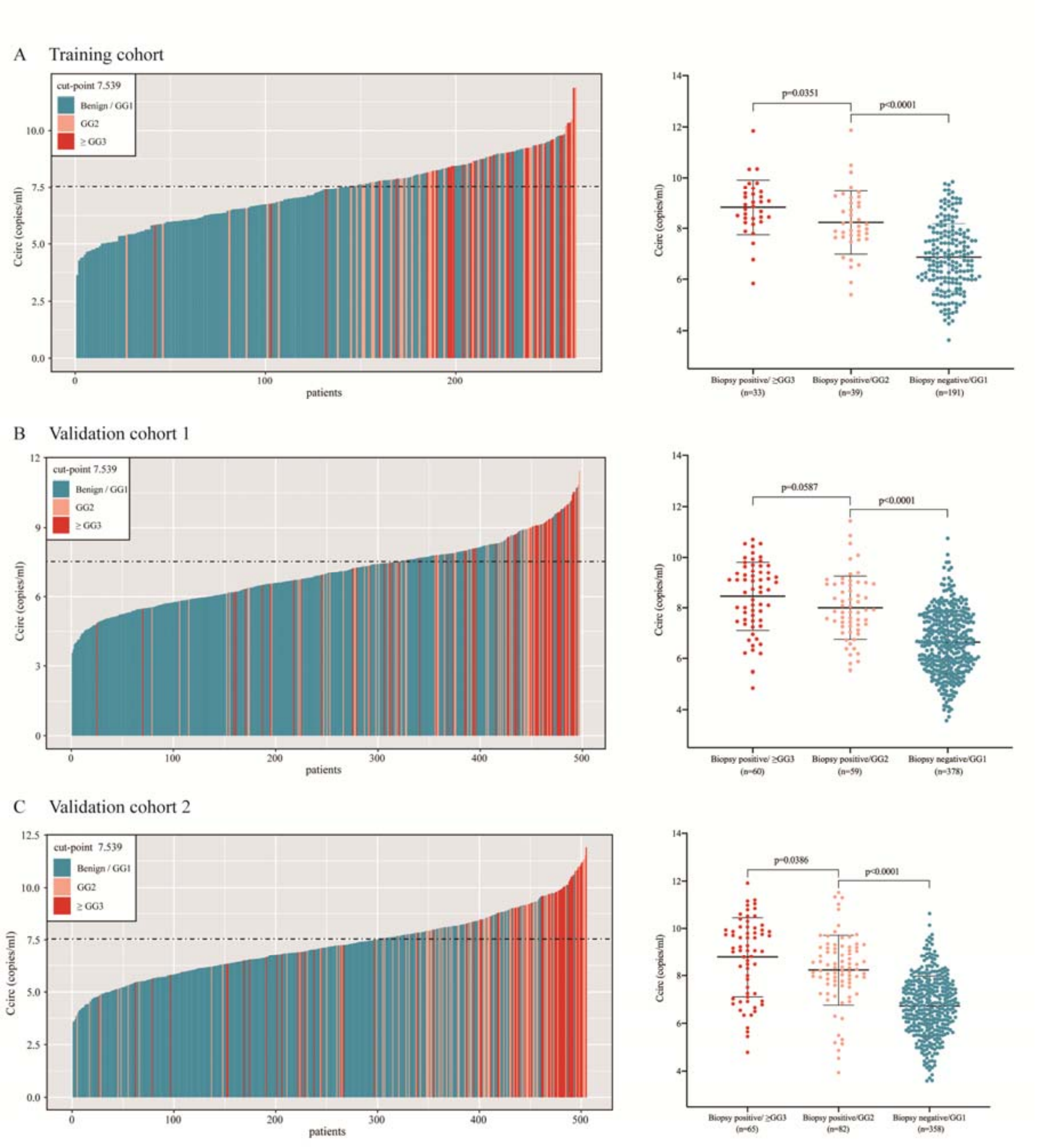

Figure S4: **The Ccirc in relation to prostate biopsy outcomes in the training and validation cohorts.**

(A-C, left) Using waterfall plot, the Ccirc is shown on *y*-axis and the Grade Group (GG) biopsy results illustrated in blue, pink, or red. **(A-C, right)** There were statistically significant differences in the Ccirc among patients with ≥GG3 PCa, GG2 PCa, and those with biopsy negative/GG1 PCa. Ccirc= classifier containing five circRNAs. GG=Grade Group. PCa=prostate cancer.

Figure S5

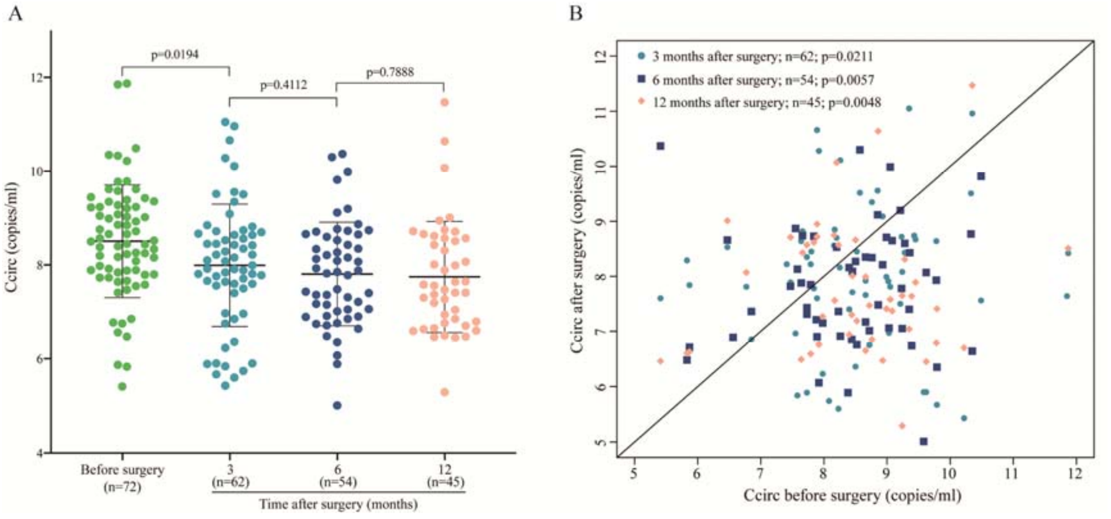

Figure S5: **The Ccirc after radical prostatectomy of high-grade PCa**

(A) The Ccirc in the high-grade PCa patients before and 3, 6, and 12 months after surgery in the training cohort. (B) Scatter plot of the Ccirc in paired urine samples from before and 3, 6, and 12 months after surgery from the same patients with high-grade PCa. Ccirc= classifier containing five circRNAs. PCa=prostate cancer.

Figure S6

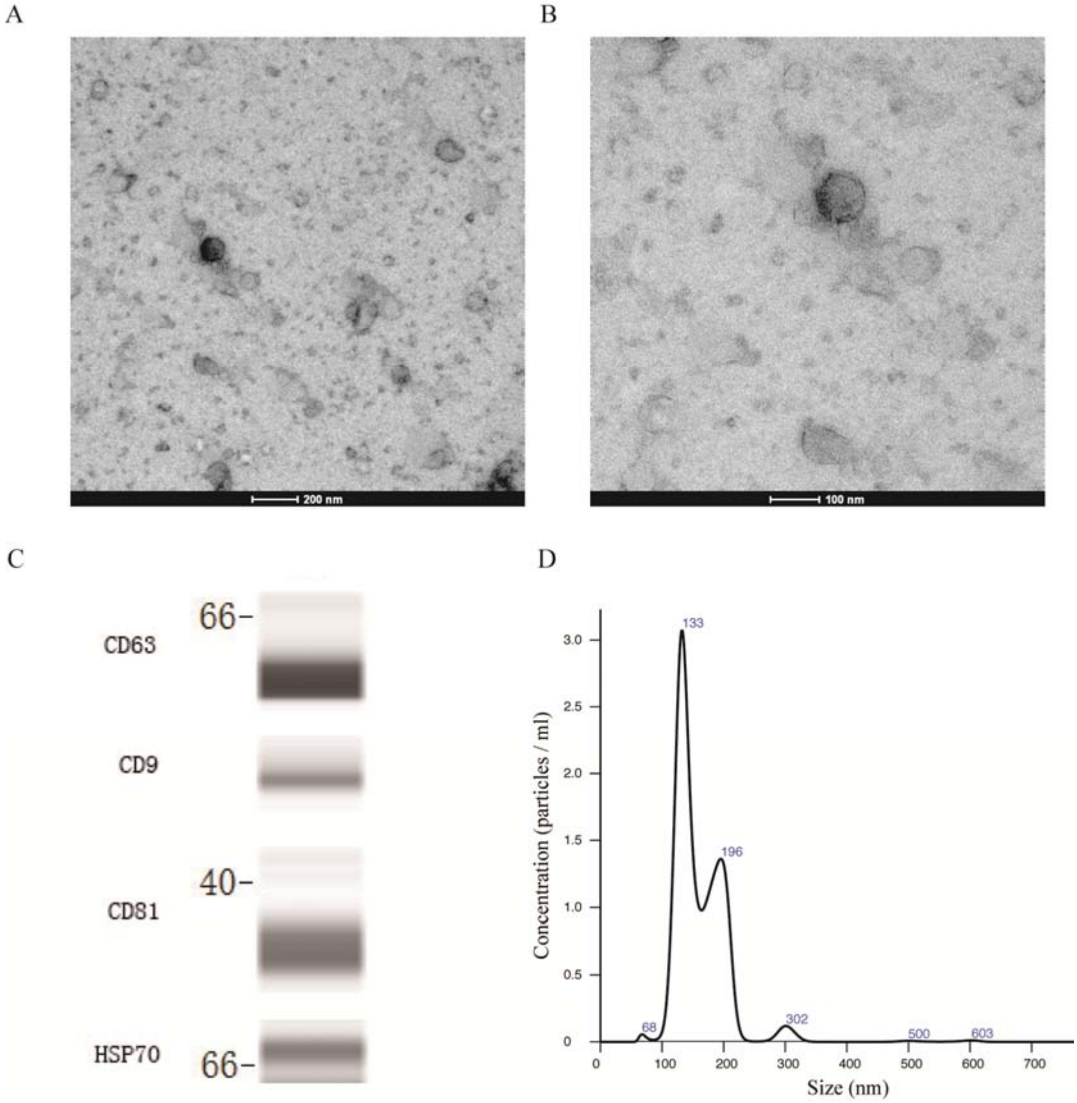

Figure S6: **Characterization of extracellular vesicles derived from human urine.** (A, B) Transmission electron microscopy images of extracellular vesicles isolated from human urine (scale bar, 200 nm and 100nm, respectively). (C) Western blotting for four representative extracellular vesicle specific markers: CD9, CD63, HSP70, and CD81. (D) The size range of urine extracellular vesicles analysed by Nanosight.

Figure S7

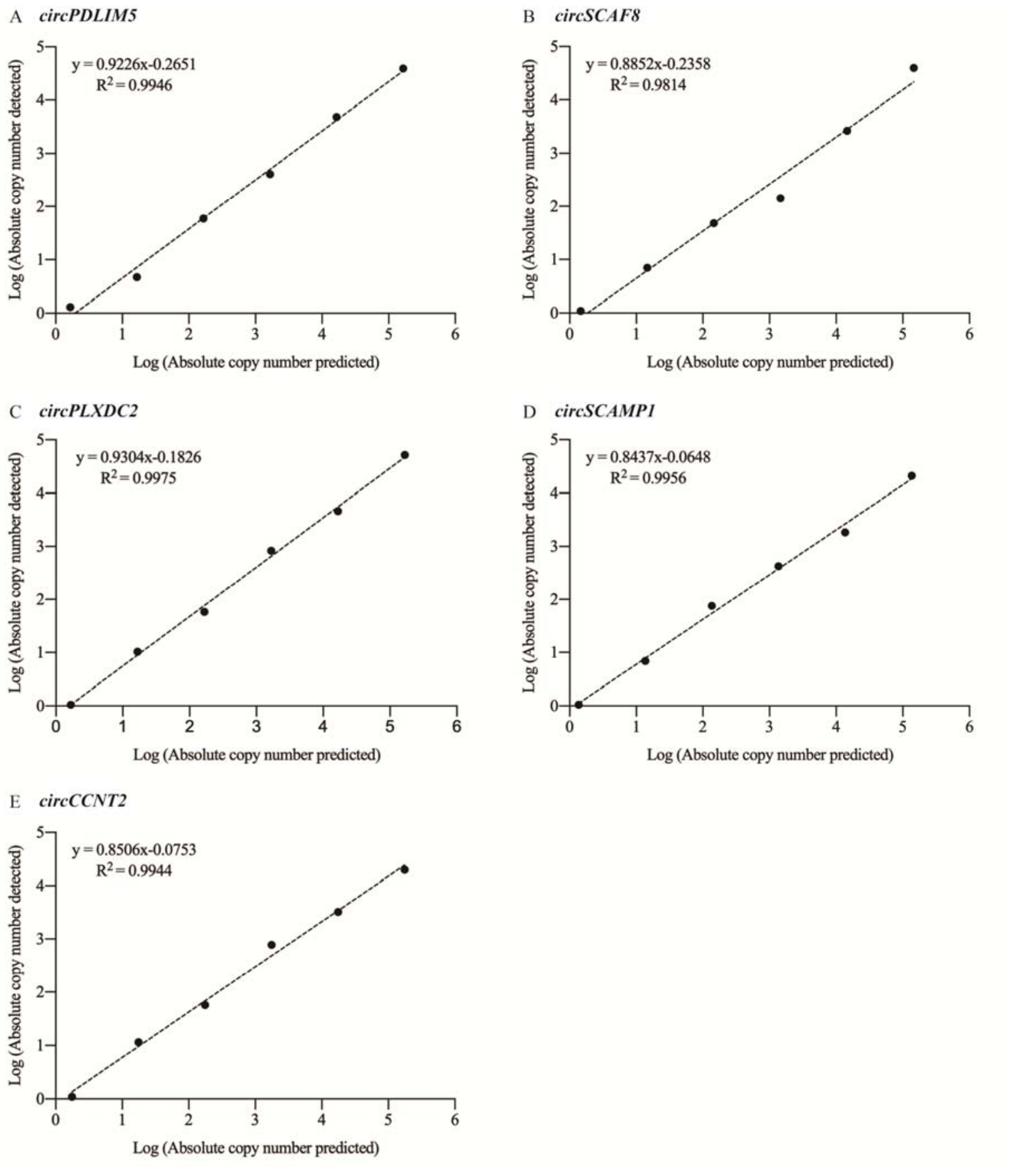

Figure S7: **Assay linearity of digital droplet PCR.**

Standard curves were conducted by 10-fold serial dilution of pUC57-circRNA vector of each circRNA, a quantification relation between the log absolute copy number detected per reaction (*y*-axis) and log absolute copy number predicted per reaction (*x*-axis). circRNA=circular RNA.

Figure S8

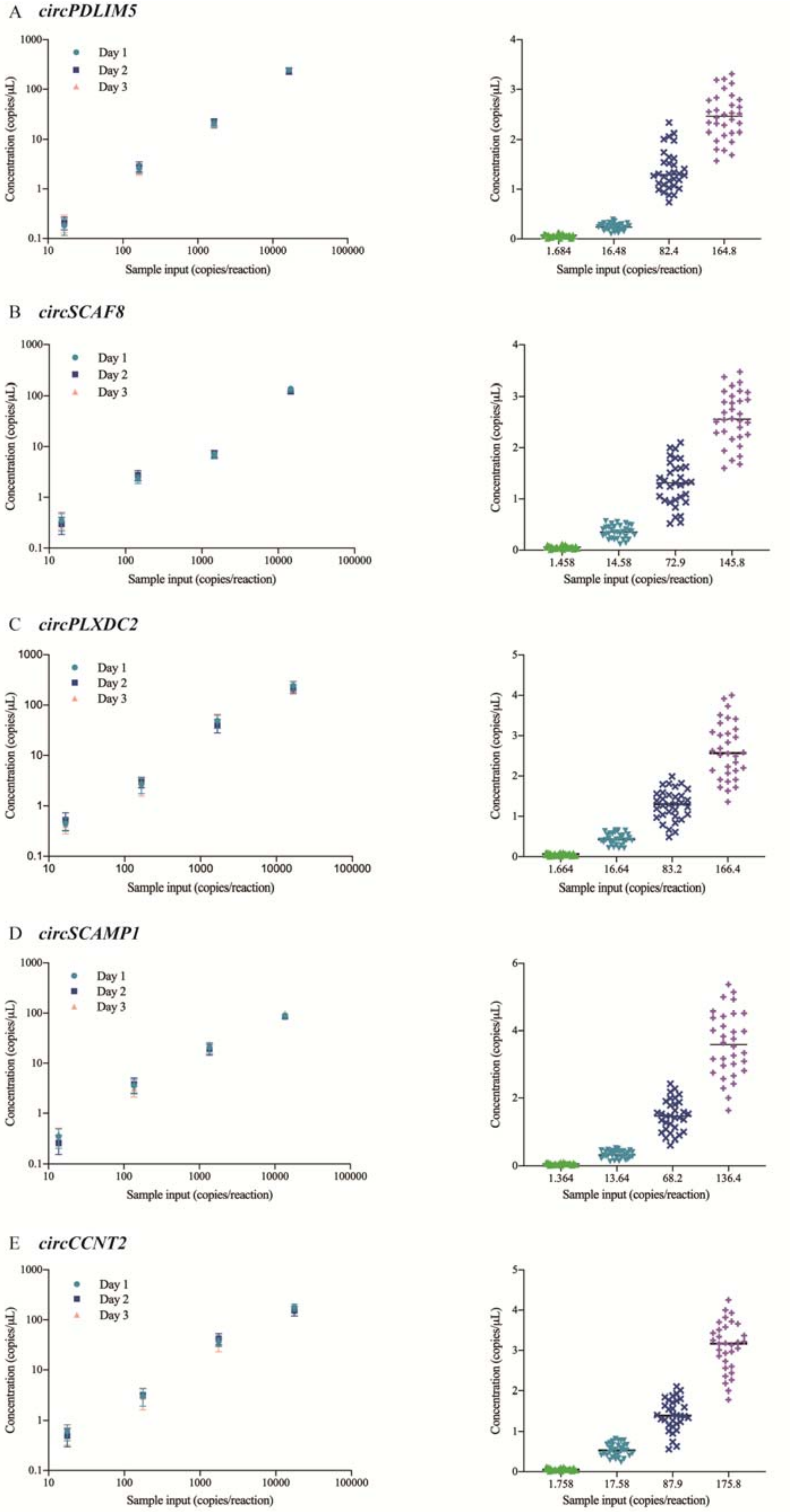
A circPDLIM5
B circSCAF8
C circPLXDC2
D circSCAMP1
E circCCNT2
Day 1
Day 2
Day 3
Concentration (copies/μL)
Sample input (copies/reaction)
1.684
16.48
82.4
164.8
1.458
14.58
72.9
145.8
1.664
16.64
83.2
166.4
1.364
13.64
68.2
136.4
1.758
17.58
87.9
175.8

Figure S8: **Inter- and intra-day variation of the digital droplet PCR assay.**

(A) The uniform 10-fold serial dilutions of each pUC57-circRNA vector were detected in triplicates on three nonconsecutive days. The percent coefficients of variation with days and between days ranged between 9.65-30.86% and 11.13-34.21%, respectively. (B) The serial dilution of pUC57-circRNA vector of each circRNA was detected 32 times on the same day and the results demonstrated that single molecule detected by digital droplet PCR followed a Poisson-distribution. circRNA=circular RNA.
